# Eosinophilic Ascites and Duodenal Obstruction in a Patient with Liver Cirrhosis

**DOI:** 10.1155/2014/928496

**Published:** 2014-02-10

**Authors:** Nasrollah Maleki, Mohammadreza Kalantar Hormozi, Mehrzad Bahtouee, Zahra Tavosi, Hamidreza Mosallai Pour, Seiiedeh Samaneh Taghiyan Jamaleddin Kolaii

**Affiliations:** ^1^Department of Internal Medicine, Imam Khomeini Hospital, Ardabil University of Medical Sciences, Ardabil, Iran; ^2^The Persian Gulf Marine Medicine Biotechnology Research Center, Department of Endocrinology, Bushehr University of Medical Sciences, Bushehr, Iran; ^3^Department of Internal Medicine, Shohadaye Khalije Fars Hospital, Bushehr University of Medical Sciences, Bushehr, Iran

## Abstract

Eosinophilic gastroenteritis (EG) is a rare disease characterized by eosinophilic infiltration of portions of the gastrointestinal tract. Eosinophilic ascites is probably the most unusual and rare presentation of EG and is generally associated with the serosal form of EG. Hereby, we report a case of eosinophilic ascites with duodenal obstruction in a patient with liver cirrhosis. A 50-year-old woman was admitted to our hospital because of abdominal pain, nausea, bloating, and constipation. She had a history of laparotomy because of duodenal obstruction 2 years ago. Based on clinical, radiological, endoscopic, and pathological findings, and given the excluding the other causes of peripheral eosinophilia, the diagnosis of eosinophilic gastroenteritis along with liver cirrhosis and spontaneous bacterial peritonitis was established. Based on the findings of the present case, it is highly recommended that, in the patients presented with liver cirrhosis associated with peripheral blood or ascitic fluid eosinophilia, performing gastrointestinal endoscopy and biopsy can probably reveal this rare disorder of EG.

## 1. Introduction

Eosinophilic gastroenteritis (EG) represents one member of a family of diseases that includes eosinophilic esophagitis, gastritis, enteritis, and colitis, collectively referred to as eosinophilic gastrointestinal disorders [1]. Despite its rarity, EG needs to be recognized by the clinician because this treatable disease can masquerade as irritable bowel syndrome. The diagnosis of EG is confirmed by a characteristic biopsy and/or eosinophilic ascitic fluid in the absence of infection by intestinal parasites or other causes of intestinal eosinophilia. The clinical features of EG are related to the layer(s) and extent of bowel involved with eosinophilic infiltration: mucosa, muscle, and/or subserosa [2]. The prevalence of each subtype is unknown because of reporting and referral biases. Surgical series report a predominance of muscular disease with obstruction [3], while medical series primarily describe patients with mucosal involvement [4, 5]. The disease can affect patients of any age, but typical presentations are in the third through fifth decade with a male predominance [4, 6].

To the best of our knowledge, this is the first reported case of eosinophilic ascites with duodenal obstruction in a patient with liver cirrhosis from Iran.

## 2. Case Presentation

A 50-year-old woman was admitted to our hospital in August 2011 because of abdominal pain, abdominal distension, nausea, bloating, and constipation. She had been experiencing recurrent episodes of urticarial skin for 20 years before her admission. There was no history of food allergies or bronchial asthma.

She had a history of intermittent abdominal pain, bloating, and constipation 3 years ago. The patient underwent an exploratory laparotomy because of acute abdominal pain and vomiting 2 years ago. During the operation, a large mass was found in the third portion of the duodenum. The mass was adherent to adjacent viscera. Also cirrhotic liver with splenomegaly was seen. There was no lymphadenopathy. The patient underwent a wedge biopsy of the cirrhotic liver and unfortunately gastrojejunostomy without removal and biopsy of the mass was done. The histopathological examination of the wedge biopsy of the liver revealed inactive cirrhosis. One year after the operation, due to the persistence of abdominal pain, bloating, and constipation, the patient was referred to our hospital.

On physical examination, her height was 159 cm, weight 82 kg, and body mass index 32.43 kg/m^2^. The patient was alert and showed no distress, was afebrile, and was hemodynamically stable. The skin and mucosa were anicteric and clear without spider angioma, and the cardiovascular and thyroid examinations were normal. Physical findings of the heart and lungs were normal. The abdomen was distended and tender diffusely with shifting dullness present; no caput medusae, rebound, or guarding was observed. Splenomegaly and bilateral leg edema were present. No lymphadenopathy was found.

Initial laboratory tests revealed a hemoglobin level of 10.5 g/dL (normal range, 12.0–16.0 g/dL), a hematocrit level of 30.1% (normal, 37.0% to 47.0%), a mean corpuscular volume of 79 fL (normal, 80.0–98.0 fL), a platelet count of 450,000/mm^3^ (normal, 150,000–450,000/mm^3^), and a white blood cell of 18,700/mm^3^ (68% neutrophils, 5% lymphocytes, and 27% eosinophils). Liver and renal functions were in normal range. Prick test results for food allergens were negative, as was a stool examination for bacteria, ova, and parasites. Tumor markers were normal. The serum immunoglobulin E level was 1,900 U/mL (normal, 6 to 90). The serum level of erythrocyte sedimentation rate was 45 mm/h, and those of viral hepatitis serology, antinuclear antibody, and antineutrophil cytoplasmic antibody were normal. There was no etiology for cirrhosis.

**Figure 1:**
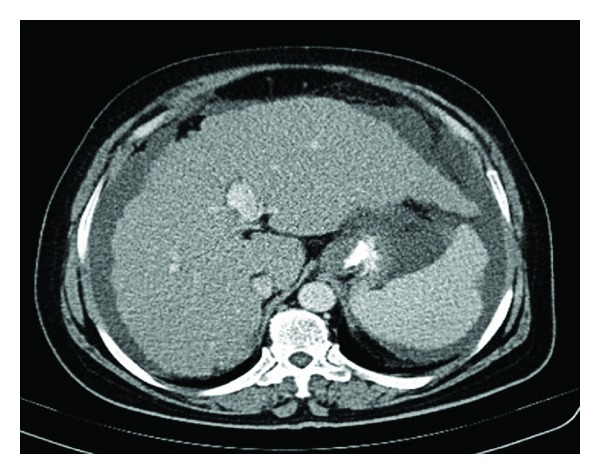
Abdominal CT scan showed a cirrhotic liver, splenomegaly, and ascites.

**Figure 2:**
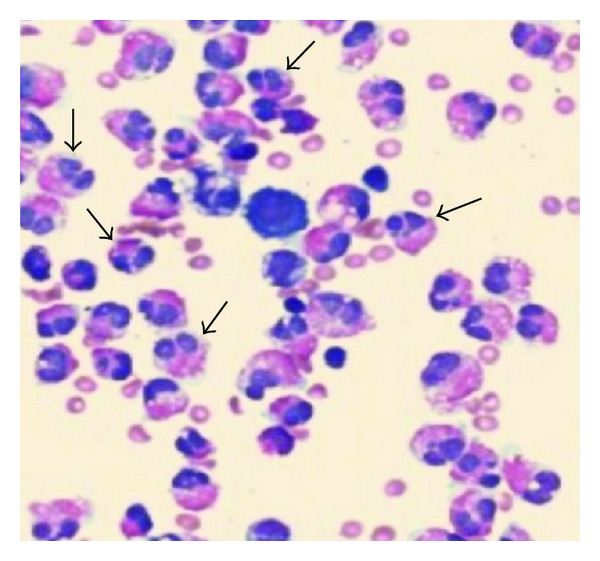
Ascitic fluid showing increased numerous eosinophils (arrows) with Giemsa stain (original magnification ×600).

Analysis of the ascitic fluid revealed a white blood cell of 3840 cu/mm (8% neutrophils, 10% lymphocytes, and 82% eosinophils) (Figure 1), a glucose 110 md/dL, a high protein content (4.15 g/dL), an elevated serum-ascites gradient (1.6 g/dL), and an adenosine deaminase 4 IU/L. There were no malignant cells on cytological examination of the ascites. In abdominal sonography, cirrhotic liver with splenomegaly, ascites, and evidence of portal hypertension were seen, but there was no tumoral mass. Abdominal computed tomography (CT) revealed a cirrhotic liver, splenomegaly, and ascites (Figure 2).

**Figure 3:**
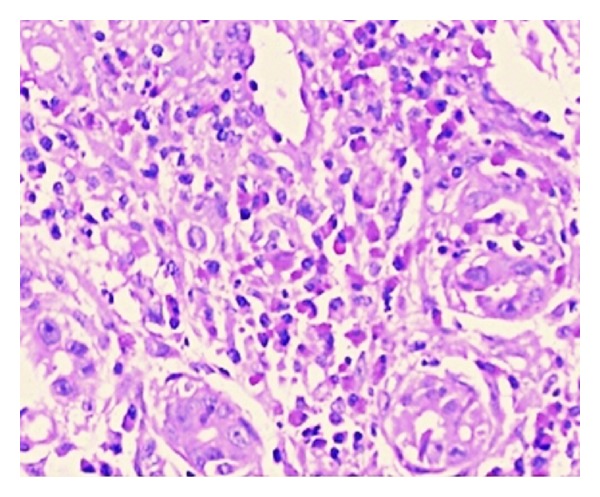
Colon biopsy shows colitis with marked eosinophilic infiltration of the colonic mucosa (H&E stain, ×100).

Upper gastrointestinal endoscopy was performed. Endoscopic examination revealed esophageal varices (grade I), evidence of moderate pangastritis, and portal hypertensive gastropathy (PHG). Location of gastrojejunostomy was visible in the antrum. Mucosal biopsies were obtained. No eosinophils in the mucosal biopsies were found. A total colonoscopy showed erythematous patches in the cecum and transverse colon, and target biopsies were performed. The final pathology report was compatible with colitis with marked eosinophilic infiltration of the colonic mucosa (Figure 3).

Based on CT, ascitic fluid analysis, colonoscopy, and pathology report findings, and given the excluding the other causes of peripheral eosinophilia, the diagnosis of eosinophilic gastroenteritis along with liver cirrhosis and spontaneous bacterial peritonitis (SBP) was established.

**Figure 4:**
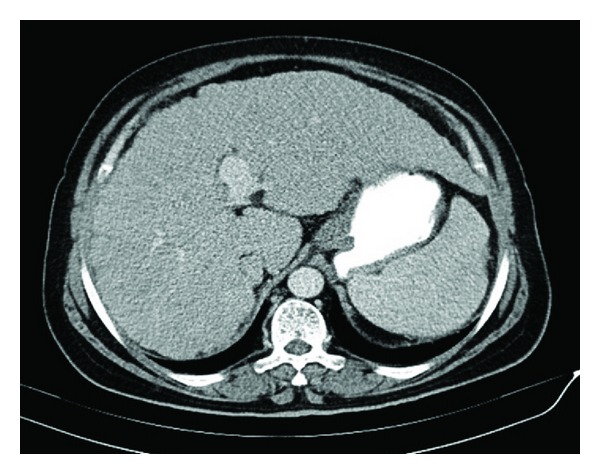
Abdominal CT scan showed complete absorption of ascites one month after treatment.

We applied prednisone 40 mg/day orally and cefotaxime (2 gr intravenous every eight hours). With this treatment, the patient's symptoms regressed, and serum white blood cell and eosinophil counts returned to normal levels after 7 days. Abdominal CT scan showed complete absorption of ascites one month after treatment (Figure 4). The prednisone dose was gradually tapered at follow-up as outpatient and successfully stopped a year later. She has remained symptom-free for more than 2 years posttreatment.

## 3. Discussion

Eosinophilic gastroenteritis (EG) is a rare, benign inflammatory disorder of the gastrointestinal tract, characterised by eosinophilic infiltration of the layers of the bowel wall, in the absence of known causes of eosinophilia [4]. EG is classified histopathologically into 3 major types: (i) predominant mucosal (60%), (ii) predominant muscle layer (30%), and (iii) predominant serosal (10%).

Eosinophilic mucosal infiltration produces nonspecific symptoms which depend upon the organ(s) involved. The entire gastrointestinal tract from esophagus to colon, including bile ducts, can be affected [4, 7–9]. The previous suggestion that EG has a predilection for the distal antrum and proximal small bowel may have reflected a sampling bias because of the availability of these areas for biopsy [2]. In a retrospective study of 40 patients, the most common symptoms were abdominal pain, nausea, vomiting, and diarrhea, suggesting a possible diagnosis of irritable bowel syndrome [4]. Only one-third of patients had a weight loss of 2.4 kg or more. Patients with diffuse small bowel disease can develop malabsorption [2, 10]. The diagnosis of mucosal EG is typically confirmed by endoscopic biopsies, which reveal ≥20 to 25 eosinophils per high power field on microscopic examination [4]. Upper endoscopy with biopsy of the stomach and small intestine is diagnostic in at least 80 percent of patients [11].

Eosinophilic infiltration of the muscle layer of the gastrointestinal tract results in a thickened, rigid gut and symptoms of intestinal obstruction such as nausea, vomiting, and abdominal distention [2, 4, 10]. Pseudoachalasia, esophageal stricture, perforation, or obstruction of the gastric outlet, small bowel, or rarely the colon can occur depending on the site of infiltration [2–4, 10]. Food intolerance or allergic history is not usually present in patients with this form of EG. Patients may present with a peripheral eosinophilia with absolute eosinophil counts averaging 1000 cells/uL [4]. The diagnosis of muscle layer disease is typically made after resection of small bowel for obstruction. Endoscopic biopsies should be performed, but they are often nondiagnostic because mucosal involvement is lacking. In these cases, laparoscopic full thickness biopsy is usually necessary to exclude malignancy, thereby avoiding unnecessary radical resectional surgery by confirming the diagnosis.

Patients with subserosal EG present with isolated ascites or ascites in combination with symptoms characteristic of mucosal or muscular EG [4]. The diagnostic feature is a marked eosinophilia, up to 88 percent, in the ascitic fluid [2]. Patients in this subgroup may have an allergic history and peripheral eosinophil counts as high as 8000 cells/uL [4]. An eosinophilic pleural effusion may also be present [10].

The pathogenesis of EG is not well understood. Approximately one-half of patients have allergic disease, such as asthma, defined food sensitivities, eczema, or rhinitis [3, 4, 12]. Some patients have elevated serum IgE levels [2, 11]. In allergic EG patients, but not those with conventional anaphylactic food allergy, a population of IL-5 expressing food allergen specific T cells have been characterized. This suggests that food exposure activates IL-5+ T cells in EG, leading to gut eosinophilia.

EG should be suspected in any patient with gastrointestinal symptoms associated with peripheral eosinophilia. It should also be considered before making a diagnosis of irritable bowel syndrome. As noted above, the diagnosis of EG can be made in almost all cases by suspicion in the appropriate clinical context and endoscopic or full thickness biopsy or paracentesis. Data on the natural history and therapy of EG are limited to case reports and retrospective series of less than 20 patients. Untreated patients with EG may rarely remit spontaneously [13] or progress to severe malabsorption and malnutrition [2]. The diagnosis of EG is based on the following 3 criteria: (1) gastrointestinal symptoms, (2) eosinophilic infiltration of 1 or more areas of the gastrointestinal tract, and (3) exclusion of other causes of intestinal eosinophilia [10].

There have been no prospective, randomized therapeutic clinical trials. Thus, treatment is empiric and based upon the severity of the clinical manifestations. Patients who are symptomatic or have evidence of malabsorption may be treated with systemic glucocorticoids. Successful transition from oral, conventional glucocorticoids to budesonide was described in patients with EG involving the gastric antrum and small intestine [14]. Oral cromolyn (800 mg/day in four divided doses) has been effective for short- and long-term management in some [15], but not all [4], case reports.

Ketotifen (Zaditen), an H1-antihistamine, has been helpful in individual cases [16, 17]. The leukotriene antagonist, montelukast, was effective in some reported cases [18] but not in others [19]. In a preliminary report of four patients, treatment with a humanized anti-interleukin-5 antibody was associated with reduced peripheral and tissue eosinophil counts but had no effect on symptoms [20]. Rebound eosinophilia has been observed after the drug was discontinued [21]. A report of nine patients treated with omalizumab described significant improvement in symptoms and measures of IgE mediated allergy [6].

## 4. Conclusion

This case report reviews some of the characteristic clinical, laboratory, and histopathological findings of a rare, readily treatable, and easily missed disease. Based on the findings of the present case, it is highly recommended that, in the patients presented with liver cirrhosis associated with peripheral blood or ascitic fluid eosinophilia, performing gastrointestinal endoscopy and biopsy can probably reveal this rare disorder of EG. Thus, awareness of this condition and timely diagnosis and initiation of treatment would be of major importance.
